# Self-Assembled BODIPY Derivative with A-D-A Structure as Organic Nanoparticles for Photodynamic/Photothermal Cancer Therapy

**DOI:** 10.3390/ijms232214473

**Published:** 2022-11-21

**Authors:** Guojing Li, Mengqian Yang, Qilong Sha, Li Li, Xiaogang Luo, Fengshou Wu

**Affiliations:** 1 Hubei Key Laboratory of Novel Reactor and Green Chemical Technology, School of Chemical Engineering and Pharmacy, Wuhan Institute of Technology, Wuhan 430072, China; 2 School of Materials Science and Engineering, Zhengzhou University, Zhengzhou 450001, China; 3 Key Laboratory of Novel Biomass-Based Environmental and Energy Materials in Petroleum and Chemical Industry, Key Laboratory for Green Chemical Process of Ministry of Education, Wuhan Institute of Technology, Wuhan 430072, China

**Keywords:** BODIPY, nanoparticles, self-assembly, photodynamic therapy, photothermal therapy

## Abstract

Organic nanomaterials have attracted considerable attention in the area of photodynamic and photothermal therapy, owing to their outstanding biocompatibility, potential biodegradability, well-defined chemical structure, and easy functionalization. However, it is still a challenge to develop a single organic molecule that obtains both photothermal and photodynamic effects. In this contribution, we synthesized a new boron-dipyrromethene (BODIPY)-based derivative (DPBDP) with an acceptor–donor–acceptor (A-D-A) structure by coupling 3,6-di(2-thienyl)-2,5-dihydropyrrolo [3,4-c] pyrrole-1,4-dione (DPP) and BODIPY. To enhance the hydrophilicity of the BODIPY derivative, the polyethylene glycol (PEG) chains were introduced to the *meso*- position of BODIPY core. The amphiphilic DPBDP was then self-assembled into related nanoparticles (DPBDP NPs) with improved hydrophilicity and enhanced absorbance in the NIR region. DPBDP NPs could simultaneously generate the singlet oxygen (^1^O_2_) and heat under the irradiation of a single laser (690 nm). The ^1^O_2_ quantum yield and photothermal conversion efficiency (PCE) of DPBDP NPs were calculated to be 14.2% and 26.1%, respectively. The biocompatibility and phototherapeutic effect of DPBDP NPs were evaluated through cell counting kit-8 (CCK-8) assay. Under irradiation of 690 nm laser (1.0 W/cm^2^), the half maximal inhibitory concentration (IC_50_) of DPBDP NPs was calculated to be 16.47 µg/mL. Thus, the as-prepared DPBDP NPs could be acted as excellent candidates for synergistic photodynamic/photothermal therapy.

## 1. Introduction

Cancer is the main disease that threatens a human being’s health [1]. The development of safe and effective treatments for cancer has received considerable attention nowadays [2,3,4,5]. Among the various cancer treatment strategies, phototherapy, including photodynamic therapy (PDT) and photothermal therapy (PTT), is a promising approach due to its safety, high efficiency, and non-invasiveness [6,7,8]. In PDT, the photosensitizer is irradiated by an appropriate wavelength of light to generate the reactive oxygen species (ROS), causing the death of tumor cells [9,10,11]. For PTT, the photothermal agent absorbs the laser energy to generate heat to thermally ablate the tumor [12,13]. The combination of PDT and PTT could enhance cancer therapy through the synergistic effect [14,15].

The chemical structure of the photosensitizers or photothermal agents plays an important role in the process of phototherapy, which directly affects its treatment efficacy. In the past few years, cyanine, rhodamines, and porphyrin derivatives have been widely used as photosensitizers in phototherapy [16,17,18,19,20,21]. Boron-dipyrromethene (BODIPY), bearing the large conjugated structure, was demonstrated as a new type of near-infrared fluorescent dye and photosensitizer in recent years due to its eminent characteristics, such as high fluorescence quantum yield, large molar extinction coefficient, good photo-stability, and efficient ROS generation [22,23,24]. However, its poor water solubility and low absorption in the near-infrared region seriously limit its potential applications in biological area [25,26]. Therefore, the introduction of hydrophilic groups and conjugated bone in molecule are generally used to overcome these limits [27,28]. In this context, here we designed and synthesized a new BODIPY-based compound (DPBDP) with a typical acceptor–donor–acceptor (A-D-A) structure, where the diketopyrrolopyrrole (DPP) acted as the donor, with the boron-dipyrromethene unit as the acceptor. The A-D-A structure in DPBDP could enhance the intramolecular charge transfer (ICT) and reduce the energy band gap, resulting in the redshift of the absorption to the near-infrared region. The covalent conjugation of 1, 8-naphthalenediimine (NDI) with BODIPY core can further promote the intramolecular charge transfer due to its strong electron withdrawing ability, and the PEG chains in NDI unit increase the hydrophilicity of the whole compound. Due to the amphiphilicity of DPBDP, it could self-assemble into the related nanoparticles (DPBDP NPs) by the reprecipitation method. DPBDP NPs exhibit high colloidal stability in aqueous solution with an average size of about 145 nm. Compared with the organic molecule, DPBDP NPs exhibit the red-shifted absorption with the maximum peak around 670 nm. In addition, DPBDP NPs can simultaneously generate ^1^O_2_ and heat under a single laser irradiation (690 nm). The low dark cytotoxicity and high photocytotoxicity of DPBDP NPs were investigated by the CCK-8 method and PI staining assay against the HeLa cells. Therefore, DPBDP NPs could be used as potential nanoagents in cancer phototherapy through a synergistic PDT/PTT treatment manner.

## 2. Results and Discussion

### 2.1. Synthesis

**Scheme 1:**
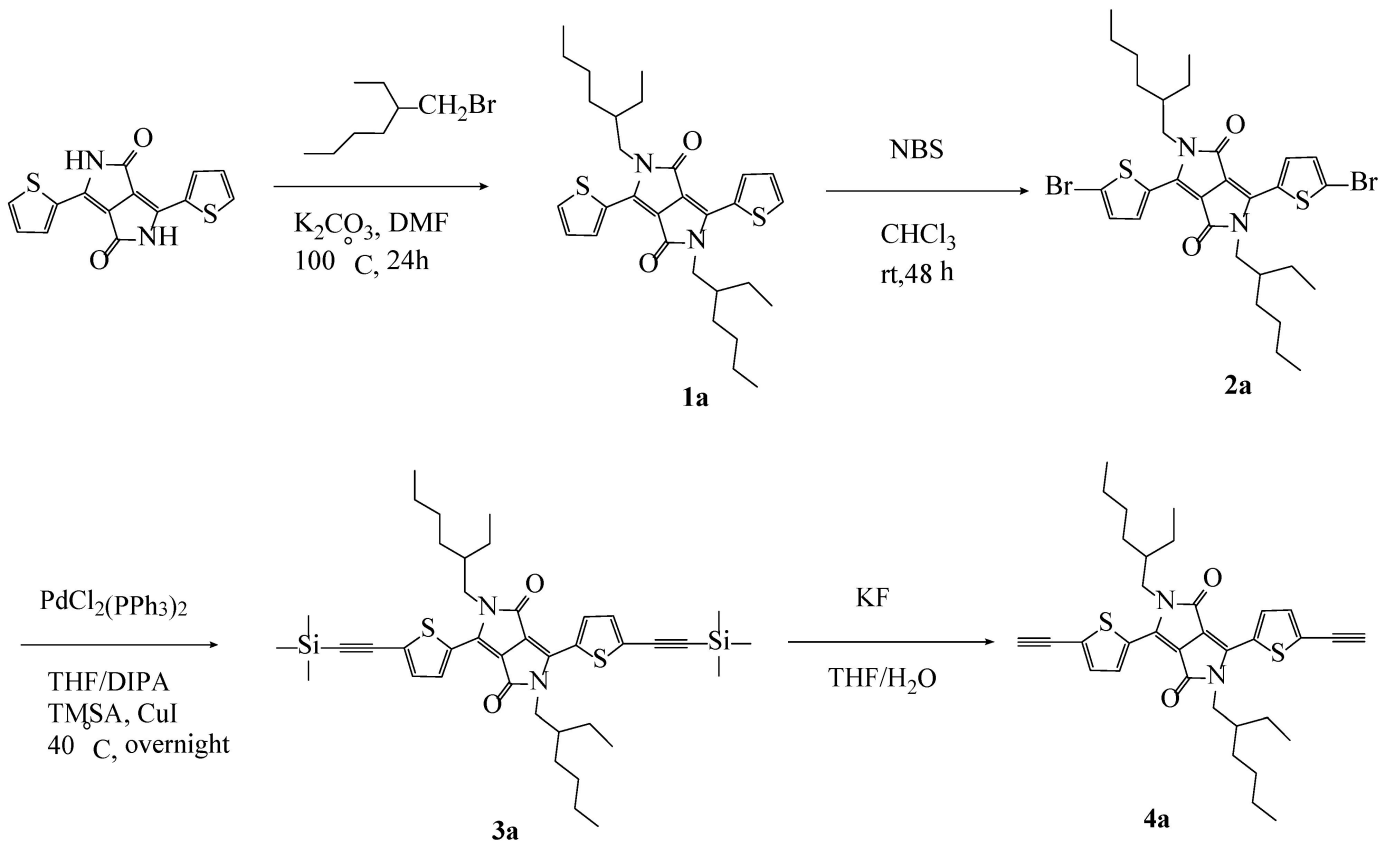
The synthetic routes of compound **4a**.

**Scheme 2:**
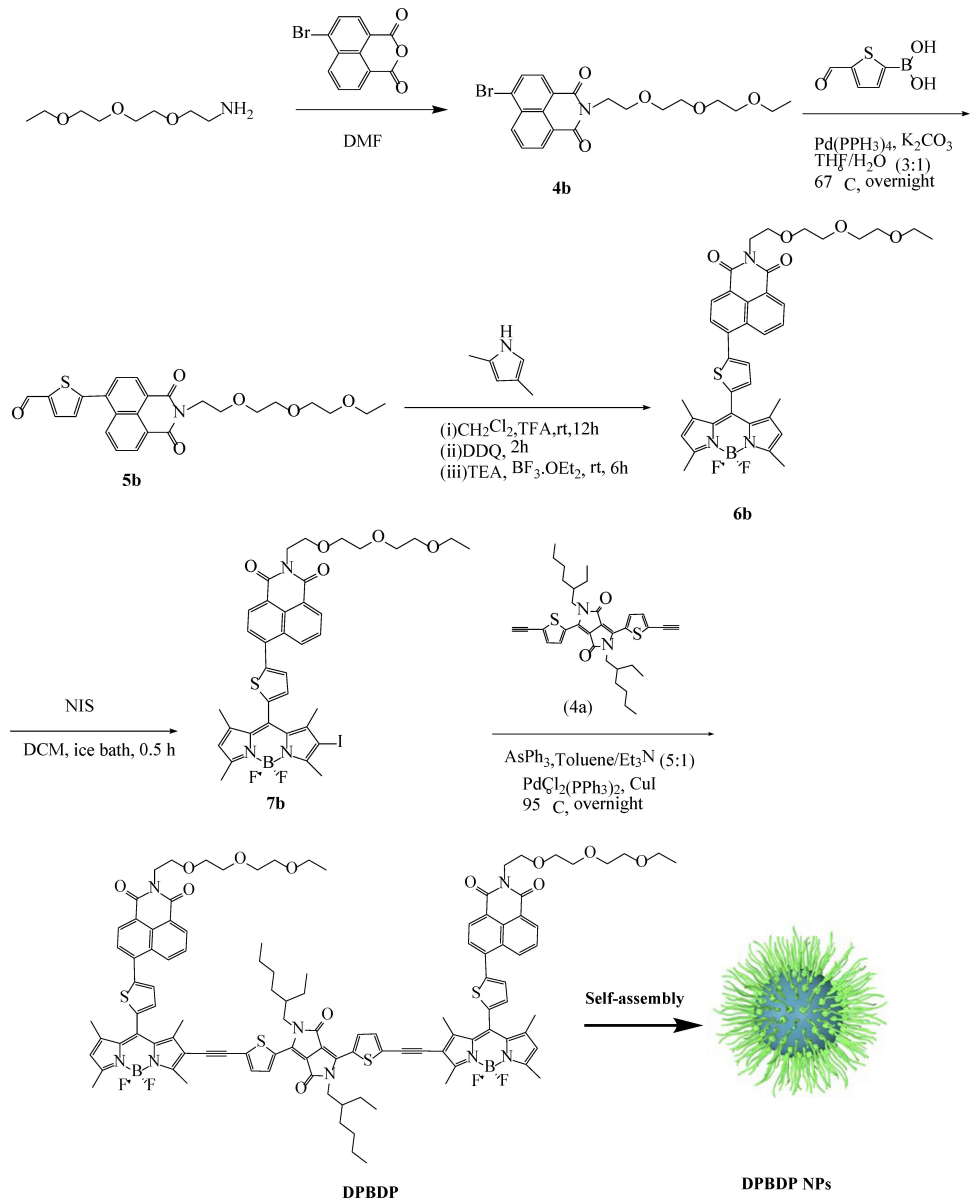
The synthetic routes of DPBDP and DPBDP NPs.

The synthetic routes of the intermediates and DPBDP NPs are shown in Scheme 1 and Scheme 2. In brief, Compound **1a** was synthesized by reacting DPP and 2-ethlhexyl bromide in DMF in the presence of K_2_CO_3_. Compound **4a** was synthesized via typical Sonogashira coupling. Compound **6b** was synthesized according to the previously reported method [29]. After iodization with N-iodosuccinimide (NIS) in CHCl_3_, the monoiodized compound **7b** was obtained in high yield [30,31,32]. Finally, DPBDP was synthesized by the conjugation of compound **4a** with compound **7b** [33]. Because of the electron-donating characteristic of diketopyrrolopyrrole (DPP) and the electron-withdrawing ability of boron-dipyrromethene, DPBDP exhibited a typical donor (D)–acceptor (A) structure, which could facilitate the intramolecular charge transfer, benefiting the π-π stacking. Moreover, the hydrophilicity of PEG chain in boron-dipyrromethene and the introduction of alkyl chains in DPP endow DPBDP with amphiphilic characteristic. The amphiphilic DPBDP could self-assemble into related nanoparticles (DPBDP NPs) through π-π stacking and hydrophobic interaction without the addition of an extra polymer, as shown in Scheme 2.

### 2.2. Theoretical Calculation

**Figure 1:**
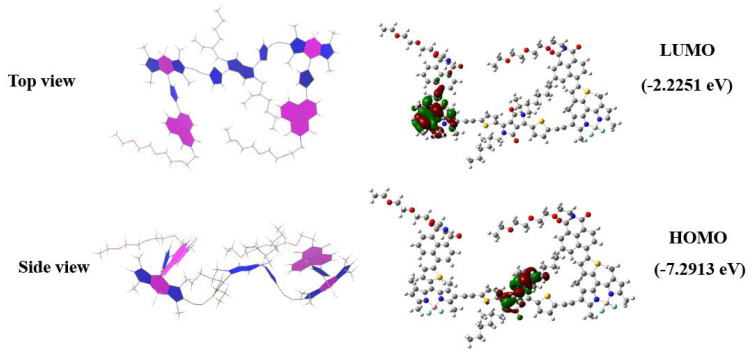
Optimized geometry and the frontier molecular orbitals electron density distribution of DPBDP.

The geometry optimizations of DPBDP were conducted through Avogadro soft. The density functional theory (DFT) calculations were investigated to obtain the electronic structures and transition energy of DPBDP with Gaussian 09 through B3LYP/sto-3g technique. As shown in Figure 1, the LUMO orbital was predominantly located on the BODIPY core, while the HOMO orbital was mainly located on the DPP unit. Accordingly, the electrons transfer from DPP to BODIPY structure would happen when DPBDP was excited to the excited states, further verifying the donor–acceptor structure of DPBDP. The HOMO energy level of DPBDP was calculated to be −7.2913 eV, while the LUMO energy levels was determined as −2.2251 eV. According to these values, the energy band gap of DPBDP was estimated as 5.0662 eV.

### 2.3. Characterization

**Figure 2:**
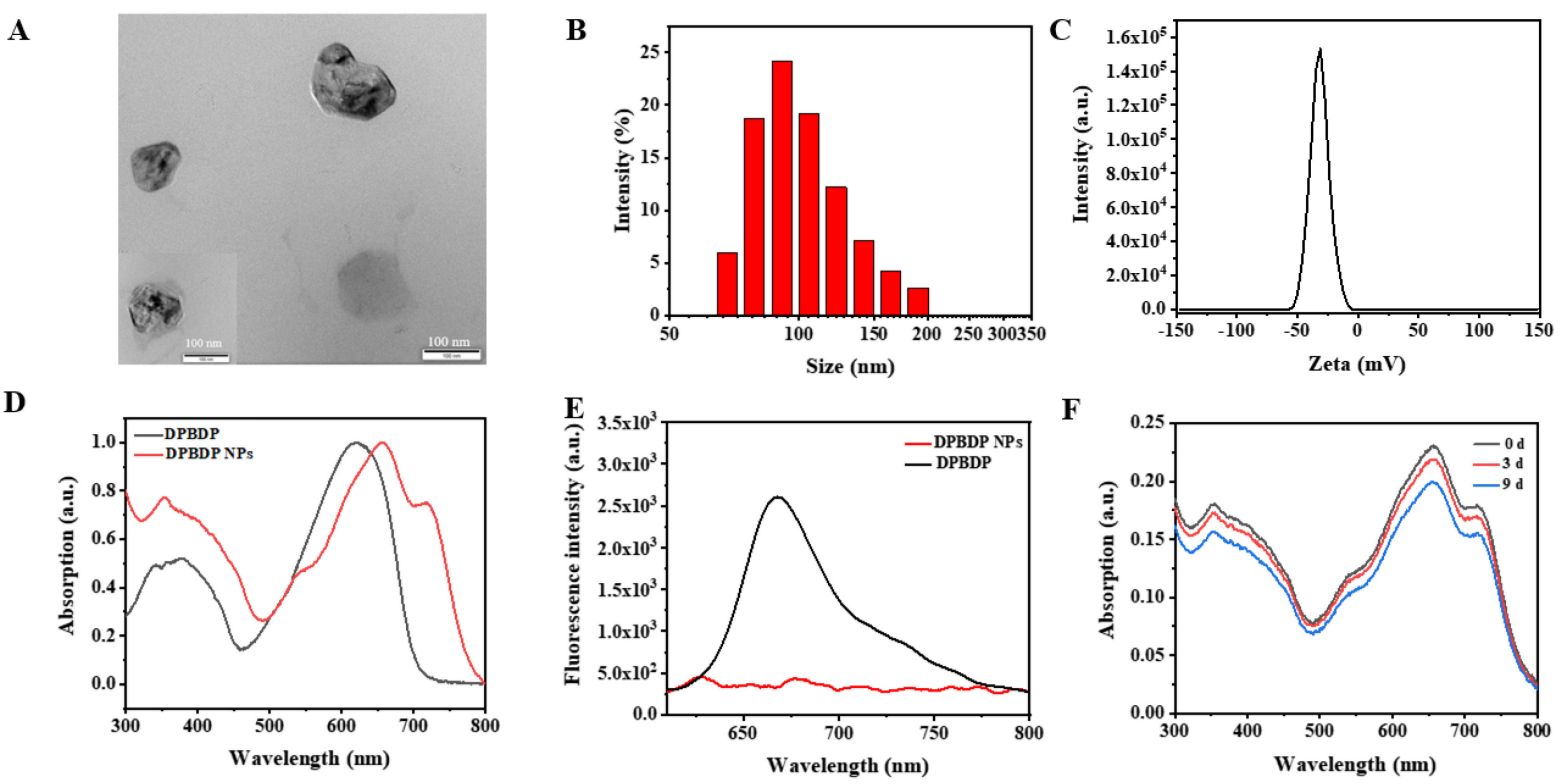
(**A**) The TEM image of DPBDP NPs, scale bar: 100 nm; (**B**) the DLS profile of DPBDP NPs; (**C**) the zeta potential of DPBDP NPs in DI water; (**D**) absorption spectra of DPBDP and DPBDP NPs; (**E**) fluorescence spectra of DPBDP, DPBDP NPs and ZnPc; (**F**) absorption spectra of DPBDP NPs in deionized water for several days.

The morphology and particle size of DPBDP NPs were described through transmission electron microscopy (TEM) and dynamic light scattering (DLS). As shown in Figure 2A, DPBDP NPs exhibited the spherical morphology with the particle size of around 140 nm. The DLS result revealed that DPBDP NPs had a reasonably uniform particle size distribution, with an average size of approximately 145 nm (Figure 2B), beneficial for the passive tumor targeting due to the enhanced permeability and retention (EPR) effect. Besides, the zeta potential of DPBDP NPs was valued as −18.3 mV (Figure 2C), which facilitated the stability of nanoparticles in aqueous medium due to the electrostatic repulsion.

### 2.4. Photophysical Properties

The photophysical data of DPBDP and DPBDP NPs are summarized in Table 1.

The UV-vis absorption spectrum of DPBDP and DPBDP NPs were examined in THF and DI water, respectively. As shown in Figure 2D, the absorption spectra of DPBDP and DPBDP NPs were similar in trend, with maximum peaks at 621 and 653 nm, respectively. Comparing with that of DPBDP, the absorption spectrum of DPBDP NPs broadened and red-shifted to some extent, probably due to the aggregation of molecules in the nanoparticles. The fluorescence spectrum of DPBDP and DPBDP NPs were investigated in the THF and aqueous solution, respectively. As shown in Figure 2E, DPBDP exhibited the strong red emission under excitation of 620 nm, with the maximum emission peak at 672 nm. However, after transforming into the nanoparticles, the fluorescence of molecule was significantly quenched, probably due to the aggregation-caused quenching (ACQ) phenomenon. The fluorescence quantum yield of DPBDP was valued to be 0.18 with ZnPc as a reference (Figure S1). The stability of DPBDP NPs in deionized water was assessed by UV-vis absorption spectrum. As shown in Figure 2F, the absorption spectrum of DPBDP NPs did not display any significant variation in a period of several days’ storage, indicating their high stability in water.

### 2.5. Reactive Oxygen Species (ROS) Generation

**Figure 3:**
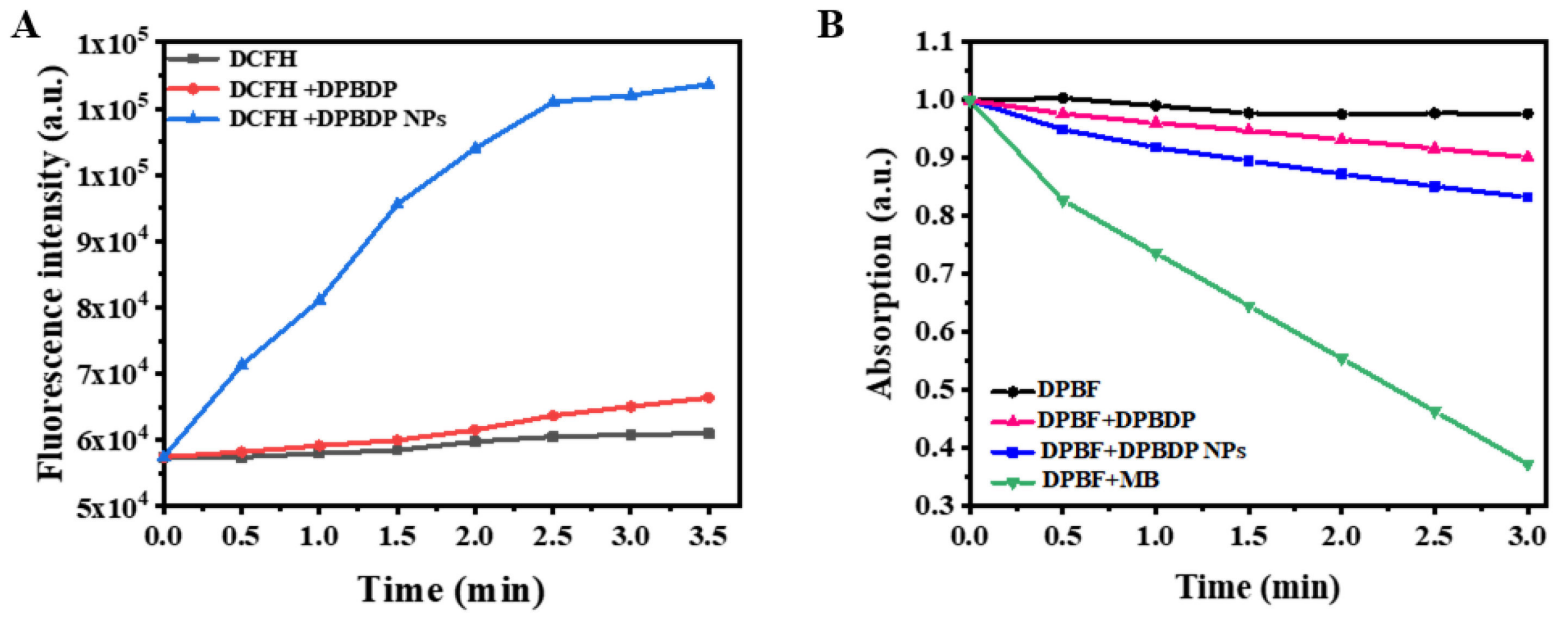
(**A**) Fluorescence intensity of DCF at 414 nm in the presence of DPBDP NPs as a function of illumination time; (**B**) linear degradation of DPBF solution with DPBDP and DPBDP NPs.

The reactive oxygen species (ROS) generation of DPBDP and DPBDP NPs was determined using 2′,7′-dichlorodihydrofluorescein diacetate (DCFH-DA) as a probe, which could be oxidized to green fluorescent DCF by ROS. As indicated in Figure 3A, the fluorescence intensity of the mixture solution of DCFH-DA and DPBDP NPs enhanced gradually with the irradiation (690 nm laser) time, which was significantly faster than that of DPBDP, indicating the higher ROS efficiency of DPBDP NPs. The singlet oxygen (^1^O_2_) generation ability of DPBDP and DPBDP NPs was further quantitatively evaluated through1,3-diphenylisobenzofuran (DPBF) method with methyl blue as standard (Figure 3B). As shown in Figure S2, the ^1^O_2_ quantum yield of DPBDP NPs (0.142) was apparently higher than that of DPBDP (0.088), which agreed well with the result of the DCFH method.

### 2.6. Photothermal Properties

**Figure 4:**
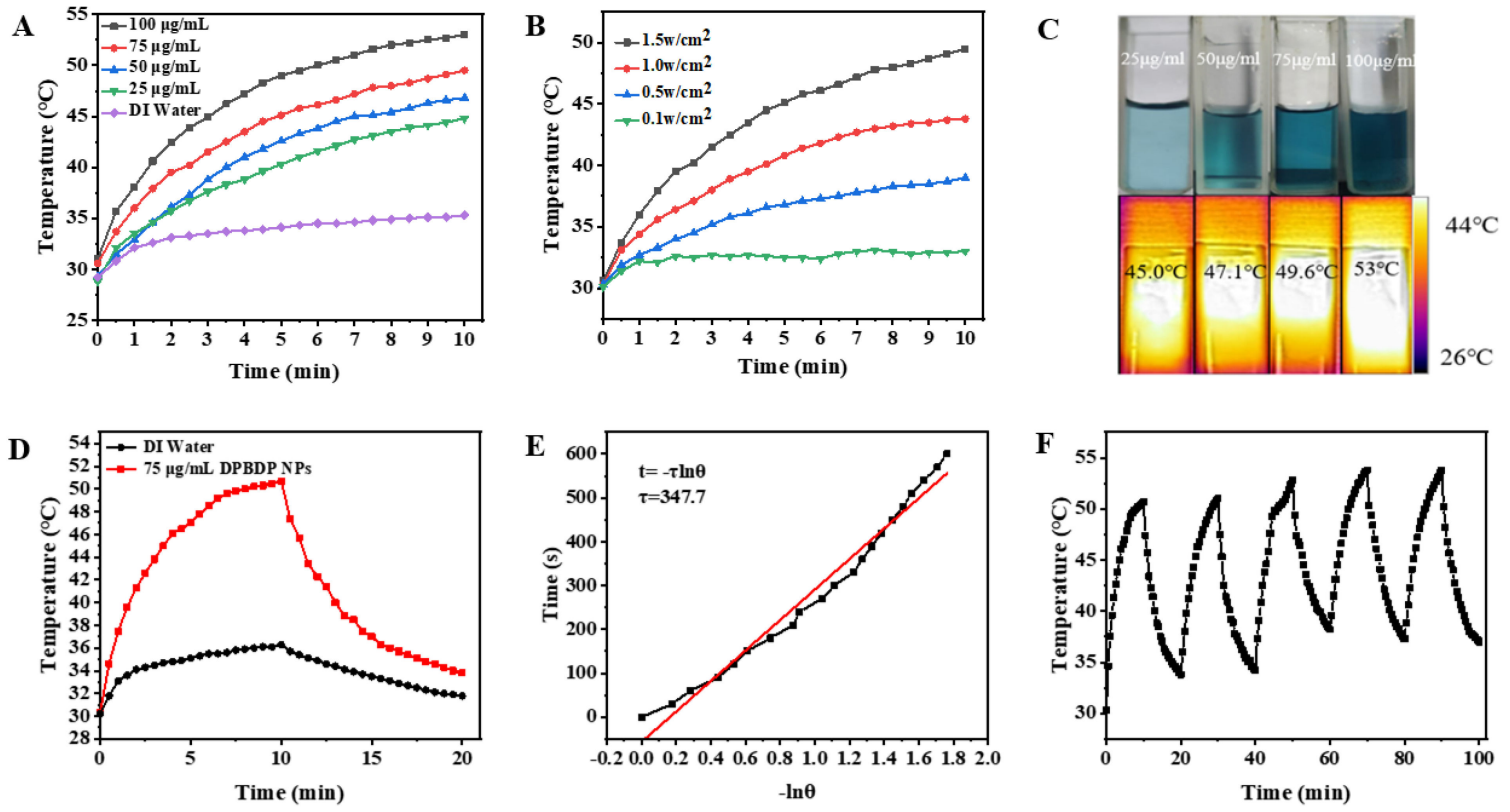
Photothermal properties of DPBDP NPs. (**A**) Photothermal curves of DPBDP NPs at several concentrations under 690 nm laser (1.5 W/cm^2^) irradiation; (**B**) photothermal curves of DPBDP NPs (75 µg/mL) under 690 nm laser irradiation at different laser power densities; (**C**) optical pictures and photothermal imaging of DPBDP NPs solution with different concentrations; (**D**) photothermal conversion effect of DPBDP NPs (75 µg/mL) and water under 690 nm irradiation; (**E**) −lnθ curve fitting of cooling time to temperature driving force in cooling phase of DPBDP NPs; (**F**) photothermal stability of DPBDP NPs during the five cycles of laser irradiation (75 µg/mL, 1.5 W/cm^2^).

The photothermal properties of DPBDP NPs were investigated by recording the temperature change of the solution at different concentrations and under laser irradiation at different power densities. As indicated in Figure 4A, the temperature rise of solution was positively correlated with the concentration of DPBDP NPs under laser irradiation (690 nm, 1.5 W/cm^2^, 10 min). Furthermore, the temperature elevation of DPBDP NPs was dependent on the laser power density (Figure 4B). The temperature elevation of the aqueous solution at different concentration was visually verified by the thermal images of the corresponding solutions after laser irradiation for 10 min (Figure 4C). The photothermal conversion efficiency (PCE) of DPBDP NPs was measured by monitoring the temperatures change of DI water and DPBDP NPs under continuous irradiation for 10 min, followed by cooling for 10 min (Figure 4D). Based on the data obtained (Figures S3 and S4), the photothermal conversion efficiency (PCE) of DPBDP NPs was calculated to be 26.1%. Moreover, the temperature change of DPBDP NPs did not display any significant variation after five cycles of heating and cooling (Figure 4E), implying the favorable photothermal stability. Therefore, DPBDP NPs could be employed as a prospective therapeutic agent for PTT.

### 2.7. Cell Viability Assay

**Figure 5:**
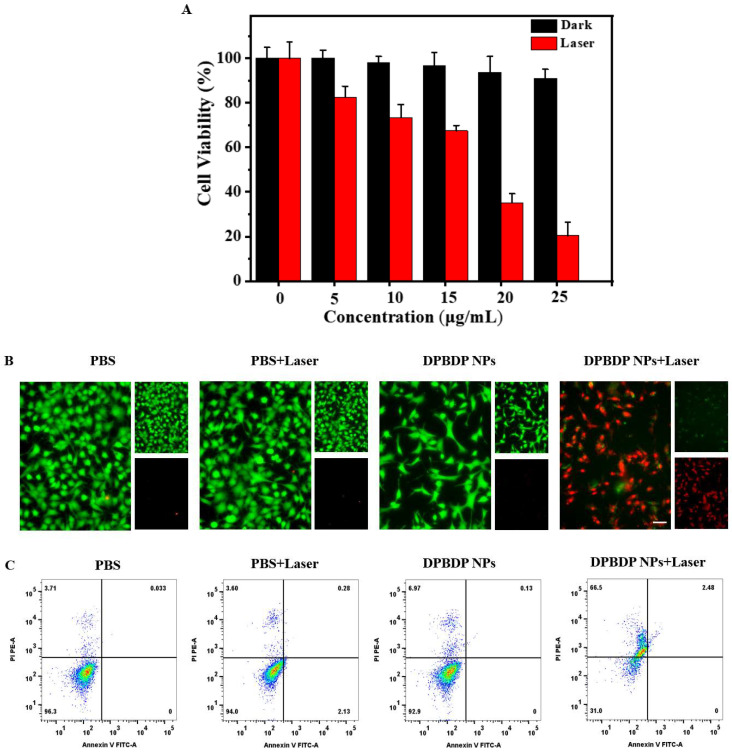
(**A**) CCK-8 assay of HeLa cells without or with 690 laser irradiation (1.0 W/cm^2^, 10 min); (**B**) calcein-AM and PI staining after 12 h incubation with PBS, PBS+laser, DPBDP NPs and DPBDP NPs+laser, scale bar = 20 µm; (**C**) cell apoptosis and necrosis level of HeLa cells stained by Annexin V-FITC/PI after 12 h incubation with PBS, PBS+laser, DPBDP NPs and DPBDP NPs+laser.

The biocompatibility and phototherapeutic effect of DPBDP NPs were evaluated through CCK-8 assay. As shown in Figure 5A and Figure S5, after incubation with DPBDP NPs in dark for 24 h, the HeLa cells still maintained over 90% viability even at the concentration of 25 µg/mL, indicating the good biocompatibility of DPBDP NPs. In contrast, under 690 nm laser irradiation (1.0 W/cm^2^) for 10 min, the cell viability decreased with the concentration of DPBDP NPs. The survival rate of cancer cells was dropped to 18% when the concentration of DPBDP NPs was elevated to 25 µg/mL, suggesting their excellent phototherapeutic efficacy. The half maximal inhibitory concentration (IC_50_) of DPBDP NPs under 690 nm laser (1.0 W/cm^2^) irradiation was calculated to be 16.47 µg/mL.

### 2.8. Live/Dead Cell Staining Assay

To further verify the biocompatibility and phototoxicity of DPBDP NPs, calcein-AM and PI staining was carried out, where the propidium iodide (PI) stains the dead cells with red color, while calcein-AM stains the living cells with green. As shown in Figure 5B, similar to the control groups (PBS and PBS+laser), the DPBDP NPs group did not display any significant red fluorescence, indicating the good biocompatibility of DPBDP NPs. However, the DPBDP NPs+laser group displayed clear red fluorescence and little green fluorescence, suggesting that the DPBDP NPs had a high phototherapeutic effect.

### 2.9. Apoptosis and Necrosis Assay

Meanwhile, the apoptosis level of HeLa cells in these groups was investigated by flow cytometer. As shown in Figure 5C, the apoptosis and necrosis percentage of cells in the DPBDP NPs+laser group was 69%, which was significantly higher than that of PBS group (3.7%), PBS+laser group (6%), and DPBDP NPs group (7.1%). Thus, DPBDP NPs presented the synergistic therapeutic effect (PTT/PDT) against HeLa cells, which was consistent with the result of the live/dead cell staining assay.

### 2.10. The Intracellular ROS Generation

**Figure 6:**
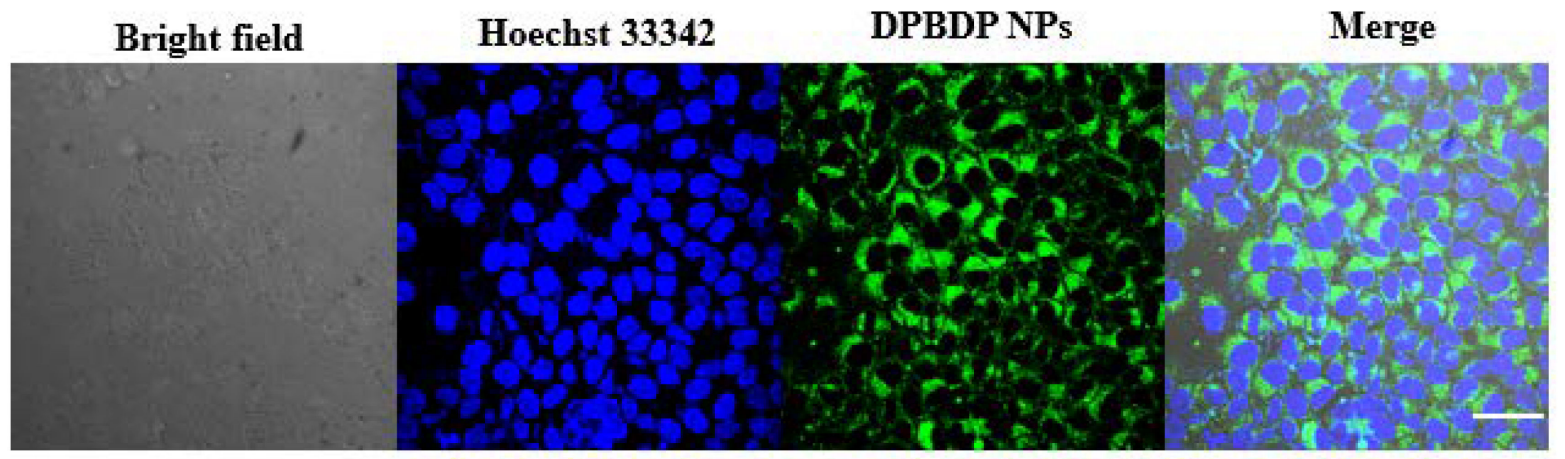
ROS generation in HeLa cells after incubation with DPBDP NPs (20 µg/mL) for 12 h followed by the treatment with DCFH-DA (10 µM) for 1 h and 690 nm laser irradiation (1.0 W/cm^2^, 5 min), scale bar = 20 µm.

The generation of ROS in cancer cells was investigated using 2′,7′-dichlorofluorescein diacetate (DCFH-DA) as a probe [38,39], which could be transformed to fluorescent 2′,7′-dichlorofluorescein (DCF) upon reacting with ROS produced by NPs under irradiation [40,41]. As shown in Figure 6, an intense green emission was observed around the nucleus of the HeLa cells, which was stained with Hoechst 33342 (blue fluorescence), suggesting the efficient intracellular ROS generation upon laser irradiation.

## 3. Experimental Sections

### 3.1. Materials and Characterization

All the starting chemicals were obtained from Aladdin (Shanghai, China) and used without further purification. The structures of intermediates and targeting materials were characterized by NMR spectra on the Agilent 400MR spectrometer, and mass spectra on a Bruker Auto flex MALDI-TOF mass spectrometer (Figures S6–S16). The absorption and fluorescence spectra were recorded through UV-vis Spectrophotometer (Shimadzu, Kyoto, Japan) and Fluorescence Spectrometer (PE LS55, Waltham, MA, USA), respectively. The DLS and zeta potential were measured by a Mavern ZetaSizer Nano-ZS (Mavern Instruments, Bejing, China). The TEM image was studied on the JEM-2100 transmission electron microscope. The excitation light was provided by a fiber-coupled 690 nm laser (MW-GX-690/700Mw, Changchun, China).

### 3.2. Synthesis of Compound **1a**

To a solution of DPP (0.9 g, 4 mmol) and anhydrous potassium carbonate (2.07 g, 15 mmol) in 40 mL of N, N-dimethylformamide, brominated isooctane (2.3 g, 12 mmol) was injected under nitrogen atmosphere. After stirring at 100 °C for 24 h, the solvent was removed by rotary evaporator. The residue was purified by silica gel column chromatography (eluent: DCM: petroleum ether = 1:1) to obtain red solid compound **1a** (1.7 g, 53%). ^1^HNMR (400 MHz, CDCl_3_) δ: 8.87 (dd, *J* = 1.0 Hz, 3.9 Hz, 2H), 7.62 (dd, *J* = 1.1 Hz, 5.1 Hz, 2H), 7.26 (dd, *J* = 3.9 Hz, 5.0 Hz, 2H), 4.01 (d, *J* = 7.6 Hz, 4H), 1.90 (m, 2H), 1.22 (m, 8H), 0.87 (m, 12H).

### 3.3. Synthesis of Compound **2a**

Compound **1a** (262 mg, 0.5 mmol) and NBS (214 mg, 1.2 mmol) were dissolved in anhydrous CH_2_Cl_2_ (20 mL). After stirring at room temperature in dark for 48 h, the reaction mixture was washed with saturated solution of Na_2_SO_3_. The organic layer was collected, and the solvent was removed under reduced pressure. The residue was purified by silica gel column chromatography (eluent: DCM: MeOH = 120:1). Compound **2a** was isolated as a red solid (192 mg, 48%). ^1^H NMR (400 MHz, CDCl_3_) δ 8.66 (d, *J* = 4.2 Hz, 2H), 7.24 (d, *J* = 4.2 Hz, 2H), 3.97–3.93 (m, 4H), 1.32 (dd, *J* = 13.6, 6.3 Hz, 18H), 0.90 (d, *J* = 7.9 Hz, 12H).

### 3.4. Synthesis of Compound **3a**

Compound **2a** (0.100 g, 0.15 mmol), CuI (2.8 mg, 0.015 mmol), PdCl_2_(PPh_3_)_2_ (5 mg, 0.0073 mmol), THF (6 mL), triethylamine (4 mL), and trimethylsilylacetylene (0.1 mL, 0.7 mmol) were added to a 50 mL Schlenk flask under nitrogen atmosphere. After being heated to 50 °C overnight, the solvent was removed under vacuum, and the crude product was further purified by silica gel column chromatography using petroleum ether/DCM (2:1) as eluent. The product was isolated as a dark purple solid with a yield of 70%. ^1^H NMR (400 MHz, CDCl_3_) δ 8.81 (d, *J* = 4.1 Hz, 2H), 7.32 (d, *J* = 4.1 Hz, 2H), 3.98 (dd, *J* = 7.7, 3.8 Hz, 4H), 1.30 (ddd, *J* = 21.1, 11.6, 6.6 Hz, 18H), 0.87 (d, *J* = 6.9 Hz, 12H), 0.27 (s, 18H).

### 3.5. Synthesis of Compound **4a**

Compound **3a** (0.1 g, 0.14 mmol), KF (0.109 g, 1.89 mmol), and deoxygenated THF/water (9 mL/2.8 mL) were added to a two-neck flask. After stirring under nitrogen atmosphere overnight, the organic fraction was extracted with dichloromethane, and the organic layer was evaporated to obtain the crude mixture, which was then purified by silica gel column chromatography using DCM/petroleum ether (7:1) as an eluent. The product was obtained in 75% yield. ^1^H NMR (400 MHz, CDCl_3_) δ 8.83 (d, *J* = 3.6 Hz, 2H), 7.38 (d, *J* = 3.5 Hz, 2H), 3.98 (s, 4H), 3.60 (s, 2H), 1.32–1.24 (m, 18H), 0.87 (d, *J* = 8.2 Hz, 12H).

### 3.6. Synthesis of Compound **4b**

To a solution of ethanol (14 mL), 4-bromo-1,8-napthalic anhydride (1.4 g, 5.1 mmol) and 2-(2-(2-methoxyethoxy) ethoxy) ethanamine 2 (1.08 g, 5.6 mmol) were added. After refluxing for 8 h, the solvent was removed under vacuum. The residue was dissolved in ethylacetate and washed with water. The organic extract was evaporated, and the crude product was purified by silica gel column chromatography (eluent: hexane: ethyl acetate =5:1) to give compound **4b** as a yellow solid (2.92 g, 90%). ^1^H NMR (400 MHz, CDCl_3_) δ 8.64 (dd, *J* = 7.3, 1.0 Hz, 1H), 8.55 (dd, *J* = 8.5, 1.0 Hz, 1H), 8.39 (d, *J* = 7.9 Hz, 1H), 8.03 (d, *J* = 7.9 Hz, 1H), 7.83 (dd, *J* = 8.4, 7.4 Hz, 1H), 4.42 (t, *J* = 6.1 Hz, 2H), 3.82 (t, *J* = 6.1 Hz, 2H), 3.69 (dd, *J* = 5.8, 3.6 Hz, 2H), 3.61 (dd, *J* = 5.9, 3.6 Hz, 2H), 3.57 (dd, *J* = 5.8, 3.8 Hz, 2H), 3.46 (dt, *J* = 14.1, 5.2 Hz, 4H), 1.16 (t, *J* = 7.0 Hz, 3H).

### 3.7. Synthesis of Compound **5b**

Under the nitrogen atmosphere, compound **4b** (870 mg, 2 mmol), 2-aldehyde phenylboronic acid (374 mg, 2.4 mmol), K_2_CO_3_ (1.1 g, 8 mmol) and Pd(PPh_3_)_4_ (185 mg, 0.16 mmol) were dissolved in 12 mL mixed solution of THF and water (THF: water = 3:1). After refluxing for 12 h, the organic solvent was removed under vacuum, and the residue was purified by silica gel column chromatography (DCM:EA = 5:1) to give compound **5b** as a yellow solid (870 mg, 70%). ^1^H NMR (400 MHz, CDCl_3_) δ 10.00 (s, 1H), 8.63 (dd, *J* = 14.4, 7.4 Hz, 2H), 8.51 (d, *J* = 8.5 Hz, 1H), 7.89 (d, *J* = 3.8 Hz, 1H), 7.84 (d, *J* = 7.6 Hz, 1H), 7.81-7.76 (m, 1H), 7.43 (d, *J* = 3.8 Hz, 1H), 4.44 (t, *J* = 6.1 Hz, 2H), 3.83 (t, *J* = 6.0 Hz, 2H), 3.71-3.68 (m, 2H), 3.63-3.60 (m, 2H), 3.57 (dd, *J* = 5.7, 3.9 Hz, 2H), 3.49- 3.42 (m, 4H), 1.15 (t, *J* = 7.0 Hz, 3H).

### 3.8. Synthesis of Compound **6b**

To a two-necked round bottom flask, compound **5b** (1 mmol), 2,4-dimethylpyrrole (2.5 mol), and anhydrous DCM (40 mL) were added under nitrogen atmosphere. After stirring at room temperature for 30 min, TFA (10 µL) was added dropwise. The resulting solution was then stirred for another 12 h followed by the addition of DDQ (1 mmol). After 2 h, 10 mL TEA was added to quench the reaction. Then, 10 mmol boron trifluoride ether was added, and the reaction mixture was stirred at room temperature for another 6 h. The solvent was then removed under vacuum, and the residue was purified by silica gel column chromatography (eluent: DCM:MeOH = 120:1) to obtain compound **6b** with a yield about 17%. ^1^H NMR (400 MHz, CDCl_3_) δ 8.62 (d, *J* = 7.6 Hz, 3H), 7.86–7.79 (m, 2H), 7.38 (d, *J* = 3.5 Hz, 1H), 7.16 (d, *J* = 3.6 Hz, 1H), 4.47 (t, *J* = 6.1 Hz, 2H), 3.86 (t, *J* = 6.1 Hz, 2H), 3.73 (dd, *J* = 5.7, 3.7 Hz, 2H), 3.64 (dd, *J* = 5.9, 3.6 Hz, 2H), 3.60 (dd, *J* = 5.8, 3.9 Hz, 2H), 3.51–3.46 (m, 4H), 2.58 (s, 6H), 1.82 (s, 6H), 1.17 (t, *J* = 7.0 Hz, 3H).

### 3.9. Synthesis of Compound **7b**

Compound **6b** (200 mg, 0.31 mmol) and NIS (229 mg, 1.24 mmol) were dissolved in anhydrous CH_2_Cl_2_ (15 mL). After stirring at room temperature for half an hour, the saturated solution of Na_2_SO_3_ was added. The organic fraction was extracted with DCM, which was then concentrated under vacuum. The obtained residue was purified by silica gel column chromatography (eluent: DCM:MeOH = 120:1) to obtain compound **7b** as an orange solid (252 mg, 92%). ^1^H NMR (400 MHz, CDCl_3_) δ 8.67–8.54 (m, 3H), 7.85–7.77 (m, 2H), 7.36 (dd, *J* = 6.3, 3.5 Hz, 1H), 7.14 (dd, *J* = 3.5, 1.2 Hz, 1H), 6.06 (s, 1H), 4.45 (t, *J* = 6.0 Hz, 2H), 3.83 (t, *J* = 6.0 Hz, 2H), 3.70 (dd, *J* = 5.7, 3.4 Hz, 2H), 3.60 (ddd, *J* = 9.5, 5.2, 3.1 Hz, 4H), 3.50–3.43 (m, 4H), 2.60 (d, *J* = 29.9 Hz, 6H), 1.80 (s, 6H), 1.15 (t, *J* = 7.0 Hz, 3H).

### 3.10. Synthesis of DPBDP

To a 25 mL flask, compound **4a** (30 mg, 0.051 mmol), **7b** (126 mg, 0.15 mmol), AsPh_3_ (200 mg, 0.523 mmol), bis-(triphenylphosphine) palladium dichloride (15 mg, 0.02 mmol), and cuprous iodide (3 mg, 0.015 mmol) were dissolved in 12 mL mixed solution of toluene and triethylamine (5:1). After stirring at 95 °C under argon overnight, the solvent was removed in vacuum. The obtained residue was purified through silica gel column chromatography (DCM:MeOH: 120:1) to yield a blue solid (17 mg, 15%). ^1^H NMR (400 MHz, CDCl_3_) δ 8.96–8.82 (m, 2H), 8.69–8.54 (m, 4H), 7.97–7.67 (m, 5H), 7.53 (s, 1H), 7.40 (s, 1H), 7.29 (d, *J* = 4.0 Hz, 1H), 7.19 (s, 3H), 6.80 (d, *J* = 8.2 Hz, 1H), 6.15 (s, 1H), 5.34 (s, 1H), 4.48–3.43 (m, 28H), 2.65 (d, *J* = 37.1 Hz, 6H), 1.89 (d, *J* = 29.4 Hz, 6H), 1.65 (d, *J* = 30.0 Hz, 8H), 1.26 (d, *J* = 13.9 Hz, 26H), 1.16 (t, *J* = 7.0 Hz, 6H), 0.92–0.83 (m, 12H). ^13^C NMR (101 MHz, CDCl_3_) δ 163.04 (s), 131.08 (d, *J* = 10.0 Hz), 128.89 (s), 128.01 (s), 127.51 (d, *J* = 12.2 Hz), 76.33 (s), 76.01 (s), 75.69 (s), 69.57 (s), 69.16 (s), 68.74 (s), 66.89 (s), 65.58 (s), 38.26 (s), 30.91 (s), 29.01 (s), 28.68 (s), 28.34 (s), 26.19 (s), 22.04 (s), 21.67 (s), 14.10 (s), 13.07 (d, *J* = 7.2 Hz), 11.86 (s), 9.47 (s). MALDI-TOF MS: calcd:1939.7345, found: 1939.7399.

### 3.11. Preparation of DPBDP Nanoparticles

DPBDP NPs were prepared through the reprecipitation approach. Specifically, DPBDP (1 mg/mL) in THF was added dropwise to DI water under sonication in a period of 15 min. The organic solvent was then removed by air blowing.

### 3.12. Photothermal Performances

The temperature of the DPBDP NPs solution at different concentrations was recorded under irradiation of a 690 nm laser (1.5 W/cm^2^, 10 min). Meanwhile, the solution temperature of DPBDP NPs (75 µg/mL) was measured upon 690 nm laser irradiation at different power densities. The PCE of DPBDP NPs was valued by comparing with DI water under irradiation of a 690 nm laser (1.5 W/cm^2^, 10 min) followed by cooling for 10 min.

### 3.13. Cells Culture and Cytotoxicity Assay

HeLa cells were cultured using Dulbecco’s Modified Eagle’s Medium (DMEM, Gibco, New South Wales, Australia) supplemented with 10% fetal bovine serum (FBS, Gibco, New South Wales, Australia) and 1% penicillin/streptomycin solution in a 37 °C cell incubator. The cell viability was evaluated through CCK-8 assay. For the cell viability assay, HeLa cells were seeded in 96-well plates (1.0 × 10^4^ cells per well) and incubated at 37 °C for 24 h. Subsequently, the cells were treated with different concentrations of DPBDP NPs (0, 5, 10, 15, 20 and 25 µg/mL) in medium. After incubation for another 12 h, the experimental cells were irradiated with or without 690 nm laser (1.0 W/cm^2^) for 10 min. The cells were then incubated for another 12 h followed by washing with PBS buffer (pH 7.4). Finally, 10% CCK-8 solution was added to the above each well followed by the incubation for 1 h. The optical density (OD) at 450 nm was measured by a Spectra Max M5 microplate reader (Molecular Devices, San Francisco, CA, USA).

### 3.14. Live/Dead Cell Staining Assay

HeLa cells were seeded in 96-well plates (3.0 × 10^3^ cells per well) and incubated at 37 °C for 24 h. Thereafter, the previous medium was replaced by fresh medium with PBS or DPBDP NPs (20 µg/mL) for 24 h. Subsequently, the experimental wells were treated with or without a 690 nm laser irradiation (1.0 W/cm^2^, 10 min). After incubation for another 24 h, the cells were stained with calcein-AM and PI for 20 min, and the cellular state was observed using an inverted fluorescence microscope (Zeiss Axio Vert.A1, Jena, Germany).

### 3.15. Apoptosis and Necrosis Assay

HeLa cells were seeded on 24-well plates (1.0 × 10^5^ cells per well) and incubated at 37 °C for 24 h. Fresh medium with PBS or DPBDP NPs were added into the plates for 24 h incubation, followed by irradiation with or without 690 nm laser for 10 min. After 24 h, the cells were stained with an Annexin V-FITC/PI apoptosis detection kit. Finally, all the treated cells were harvested and analyzed by flow cytometer (FCM, BD FACSVerse, Piscatway, NJ, USA).

### 3.16. The Intracellular ROS Generation

HeLa cells were plated into confocal dishes. After incubation for 24 h, the cells were co-incubated with DPBDP NPs (20 µg/mL). After 24 h, the medium was removed followed by washing twice with PBS. DCFH-DA (10 µM) in medium was then added. After incubation for 30 min, the cells were irradiated under 690 nm laser (1.0 W/cm^2^) for 5 min, followed by washing twice with PBS. The cells were then stained with Hoechst 33342, and the fluorescent images were collected through a confocal laser scanning microscopy (Zeiss LSM780, Jena, Germany).

## 4. Conclusions

In summary, a new BODIPY-based derivative (DPBDP) with an acceptor–donor–acceptor (A-D-A) structure was synthesized by coupling the DPP unit with BODIPY core. To enhance the hydrophilicity of the BODIPY derivative, the PEG chains were introduced to the *meso*- position of BODIPY core. The amphiphilic DPBDP was then self-assembled into related nanoparticles (DPBDP NPs) with improved hydrophilicity and enhanced absorbance in the NIR region. Compared with the organic molecules, DPBDP NPs exhibited the red-shifted absorption with the maximum peak around 670 nm. DPBDP NPs exhibited efficient photodynamic and photothermal effects under irradiation of 690 nm laser. The ^1^O_2_ quantum yield and PCE of DPBDP NPs were calculated to be 14.2% and 26.1%, respectively. The good biocompatibility and remarkable phototoxicity of DPBDP NPs were verified through the CCK-8 method against HeLa cells. The IC_50_ of DPBDP NPs under irradiation was calculated to be 16.47 µg/mL Therefore, DPBDP NPs could be used as potential nanoagents in cancer phototherapy through a synergistic PDT/PTT treatment manner.

## Figures and Tables

**Table 1:** The photophysical properties of DPBDP and DPBDP NPs.

Sample	Absorption Peaks (nm)	ε (L·moL^−1^·cm ^−1^)	Emission Peaks (nm)	Φ_f_^a^	Φ_Δ_^b^
DPBDP	621	3.0 × 10^5^	672	0.18	0.088
DPBDP NPs	653	3.3 × 10^5^	--	---	0.142

^a^ The fluorescence quantum yields (Φ_f_) were determined using zinc phthalocyanine as a reference [34,35]; ^b^ the singlet oxygen quantum yields (Φ_Δ_) were computed using 1,3-diphenylisobenzofuran (DPBF) as a capture reagent and methylene blue (MB) as a reference under 690 nm laser irradiation [36,37].

## Supplementary Materials

The following supporting information can be downloaded at: https://www.mdpi.com/article/10.3390/ijms232214473/s1
