# Supplementary material for: Self-Assembled BODIPY Derivative with A-D-A Structure as Organic Nanoparticles for Photodynamic/Photothermal Cancer Therapy
Source: Int J Mol Sci. 2022 Nov 21;23(22):14473. doi: 10.3390/ijms232214473 (PMC9698044; doi:10.3390/ijms232214473)
Supplement: Supplementary file 1 [file ijms-23-14473-s001.zip › ijms-1888703-supplementary.pdf]

# Supplementary Information

## Self-assembled BODIPY derivative with A-D-A structure as organic nanoparticles for photodynamic/photothermal cancer therapy

Guojing Li [1], Mengqian Yang [1], Qilong Sha [1], Li Li [1], Xiaogang Luo [1,3], Fengshou Wu [1,2*]

[1] Faculty of Resources and Environmental Engineering, Guizhou Institute of Technology, \linebreak Guiyang 550003, China

[2] State Key Laboratory of Environmental Geochemistry, Institute of Geochemistry, Chinese Academy of Sciences, Guiyang 550081, China.

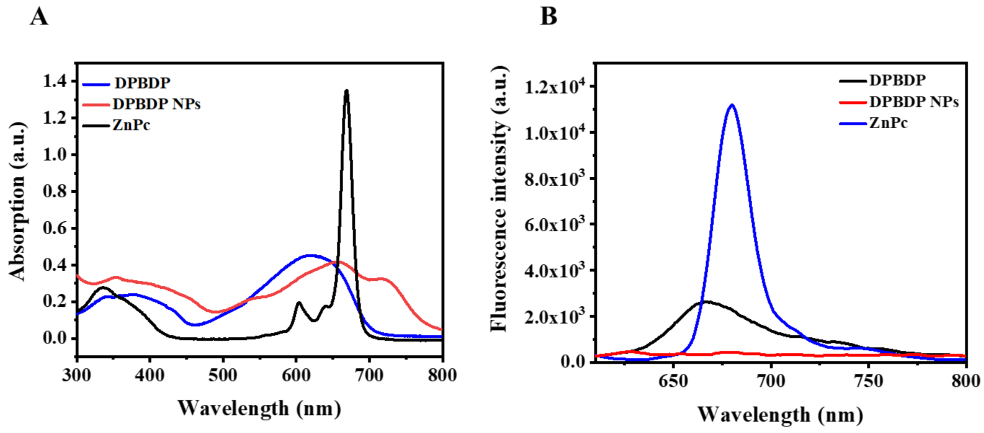


**Figure S1**. (A) Absorption spectrum of DPBDP in THF, DPBDP NPs in deionized water and ZnPc in toluene at the same concentration; (B) The fluorescence spectra of the same concentration of DPBDP, DPBDP NPs, and ZnPc.

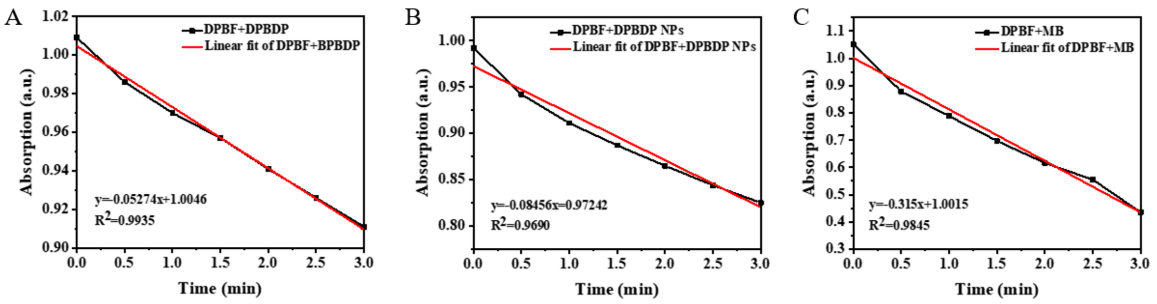


**Figure S2.** The fitting curve of absorbance with time (A) DPBF+DPBDP; (B) DPBF+DPBDP NPs; (C) DPBF+MB

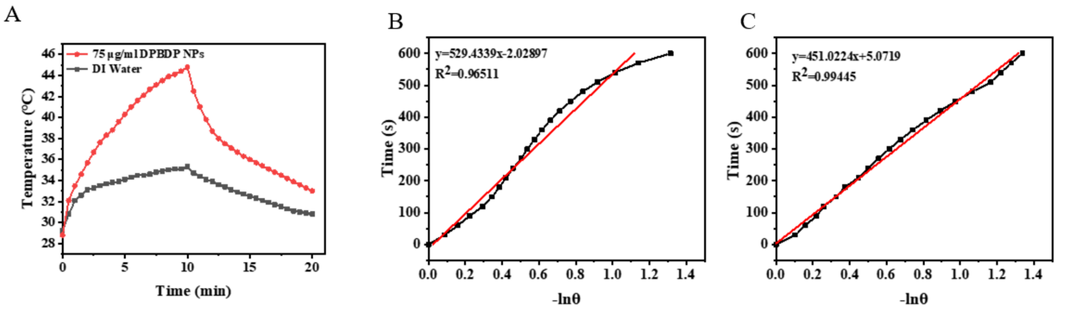


**Figure S3**. (A) Photothermal curves of DPBDP NPs (75 μg/mL) and deionized water under laser irradiation (690 nm, 1.5 W/cm$^2$, 10 min) followed by naturally cooling; -lnθ curve fitting of cooling time to temperature driving force in cooling phase of DPBDP NPs (75μg/mL) (B) and DI water (C).

## The calculation of fluorescence quantum yield

The fluorescence quantum yields of DPBDP and DPBDP NPs were calculated using the following equation with zinc phthalocyanine (ZnPc) as a reference.

$$\varphi_f = \varphi_f^R \frac{I_f}{I_f^R} \frac{A^R}{A} \left(\frac{n}{n^R}\right)^2$$

$\varphi_f^R$ is the fluorescence quantum yield of ZnPc (0.30, in toluene); The letters *S* and *R* represent the samples and ZnPc respectively; *A* is the absorbance; *I* is the integrated fluorescence intensity, and the samples and ZnPc were excited at the same wavelength for measuring *I*; *n* is the solvent's refractive index.

**Singlet oxygen detection**

The experiments were performed with 50 μM DPBF in MeOH/H2O (1:1), followed by the addition of photosensitizer (0.5 μM). The cuvette was irradiated under 635 nm laser at 0.05 W/cm$^2$. The change of absorbance at 414 nm was recorded every 30 seconds. The $^1O_2$ yields of DPBDP and DPBDP NPs were calculated by the following equation.

$$\Phi_{\Delta}^{S} = \Phi_{\Delta}^{R} \frac{k^S F^R}{k^R F^S}$$

$\Phi_{\Delta}^{R}$ is the singlet oxygen quantum yield of MB ($\Phi_{\Delta}^{R}$ = 0.52). The letters *S* and *R* represent the samples and MB respectively; *k* is the slope of the curve of DPBF absorbance (414 nm) versus irradiation time. *F* is the absorption correction factor ($F$=1–$10^{-OD}$). *OD* is the corresponding absorbance of samples at 690 nm.

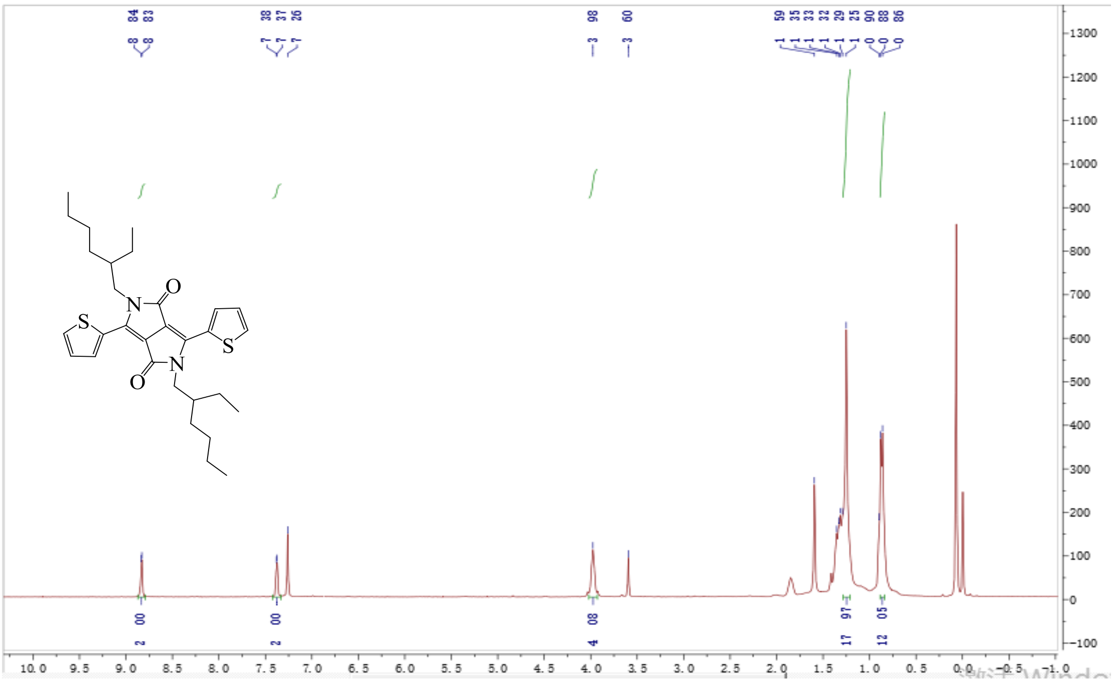

**Figure S4**. $^{1}$H NMR spectrum of compound 1a

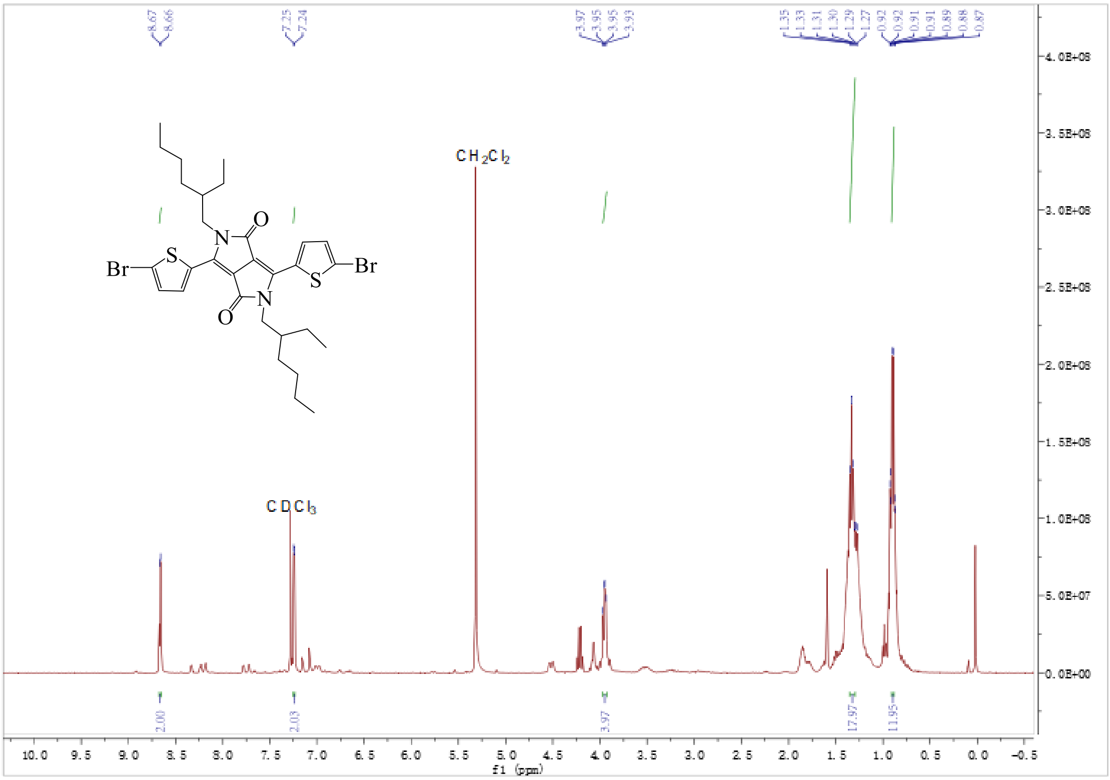


**Figure S5**. $^{1}$H NMR spectrum of compound 2a

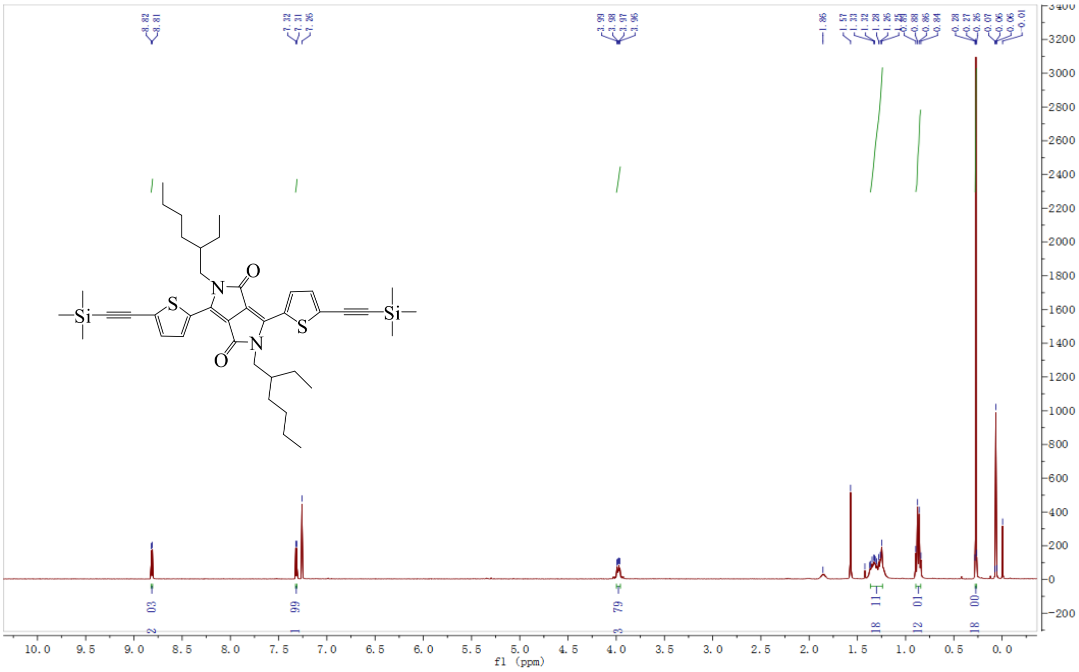

**Figure S6**. $^{1}$H NMR spectrum of compound 3a

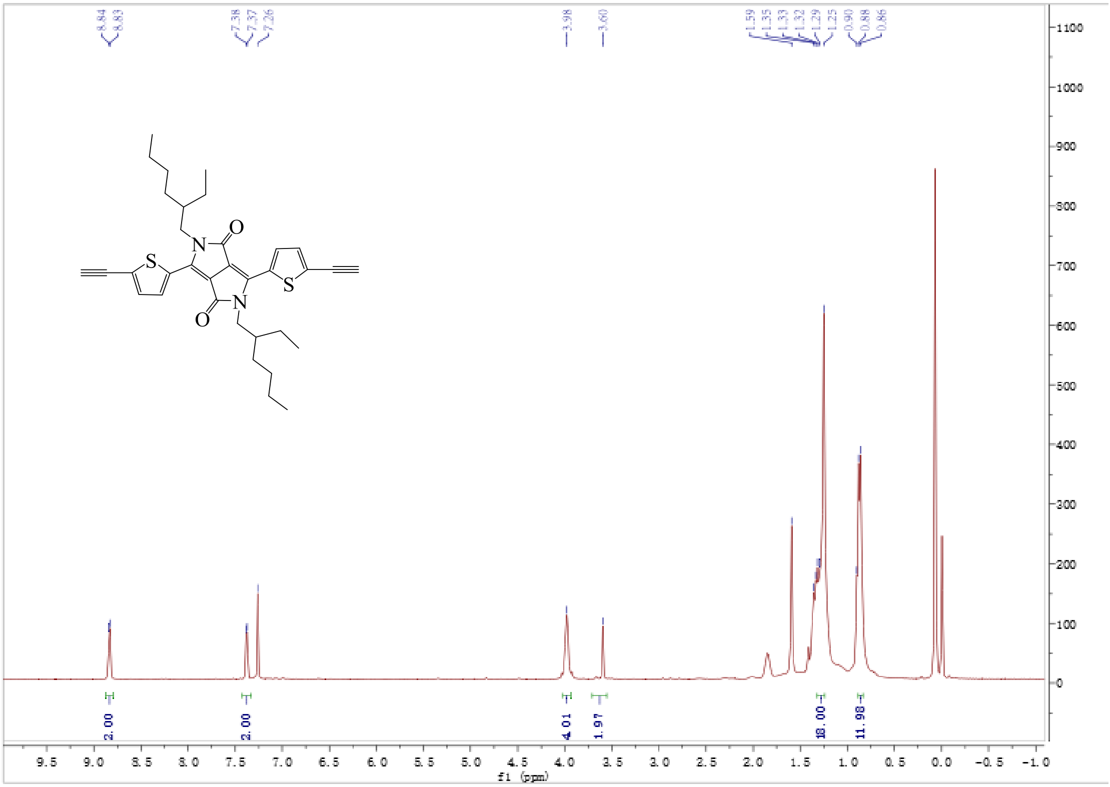

**Figure S7**. $^{1}$H NMR spectrum of compound 4a

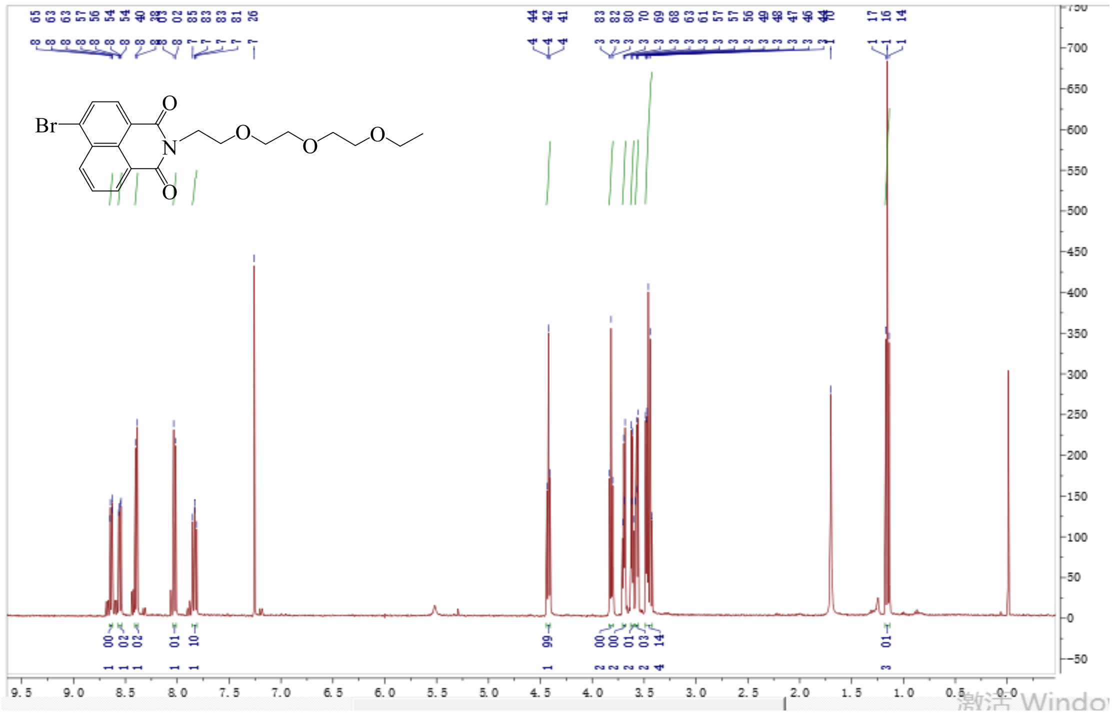

**Figure S8**. $^{1}$H NMR spectrum of compound 4b

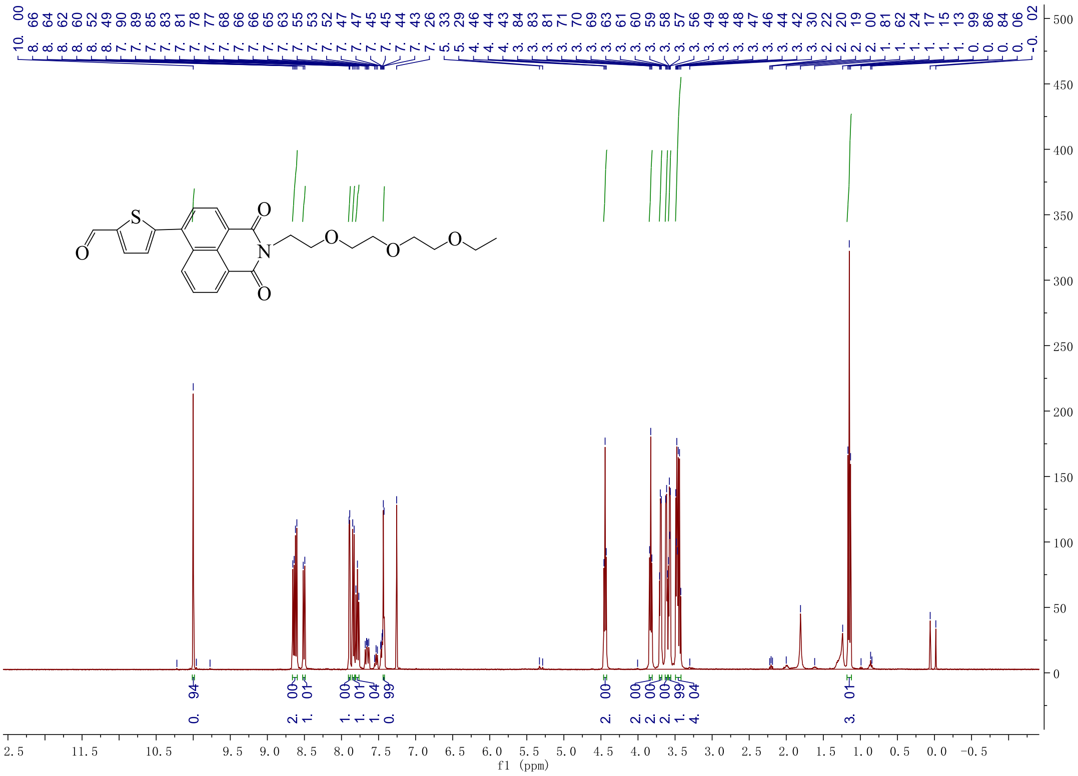

**Figure S9**. $^{1}$H NMR spectrum of compound 5b

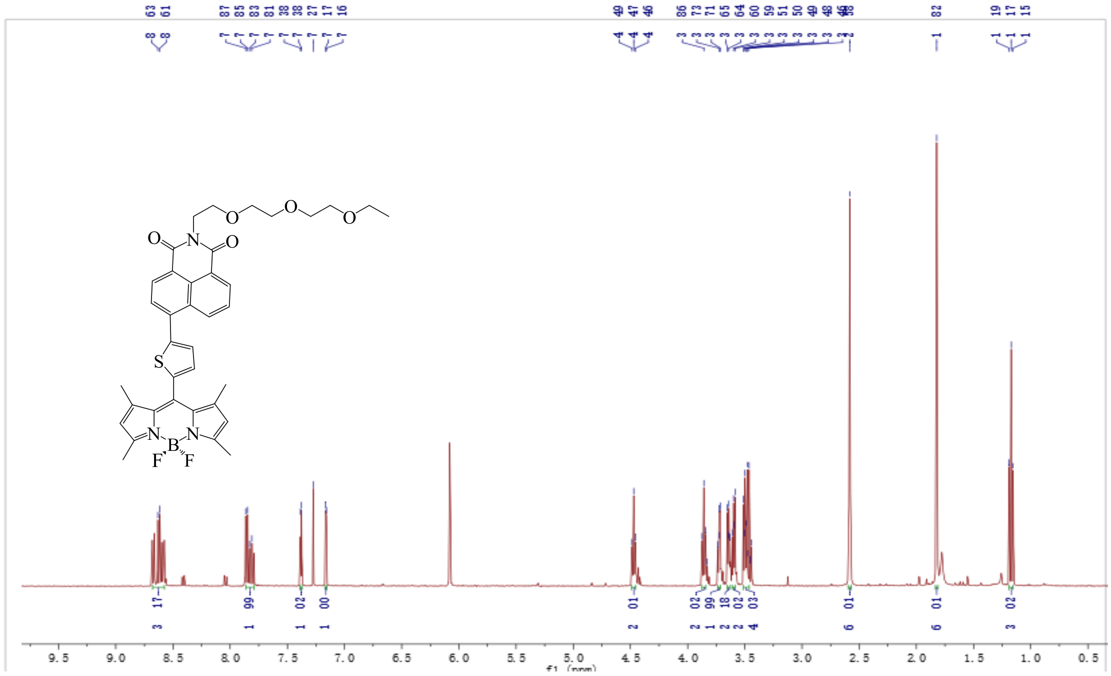

**Figure S10**. $^{1}$H NMR spectrum of compound 6b

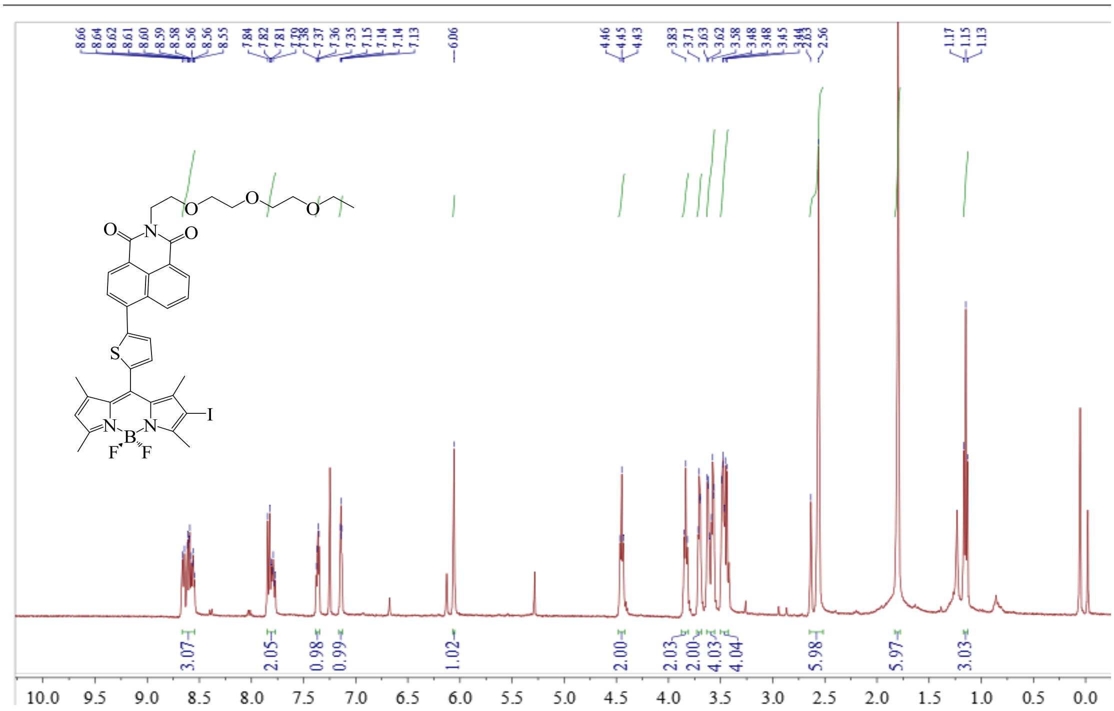

**Figure S11**. $^{1}$H NMR spectrum of compound 7b

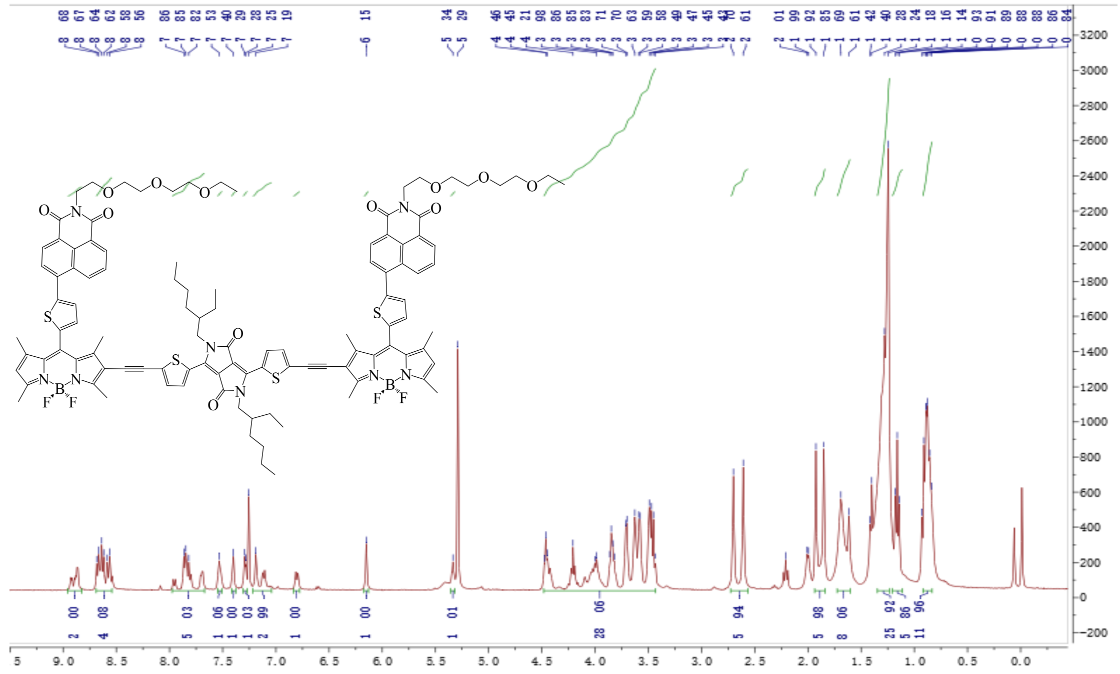


**Figure S12**. $^{1}H$ NMR spectrum of compound DPBDP

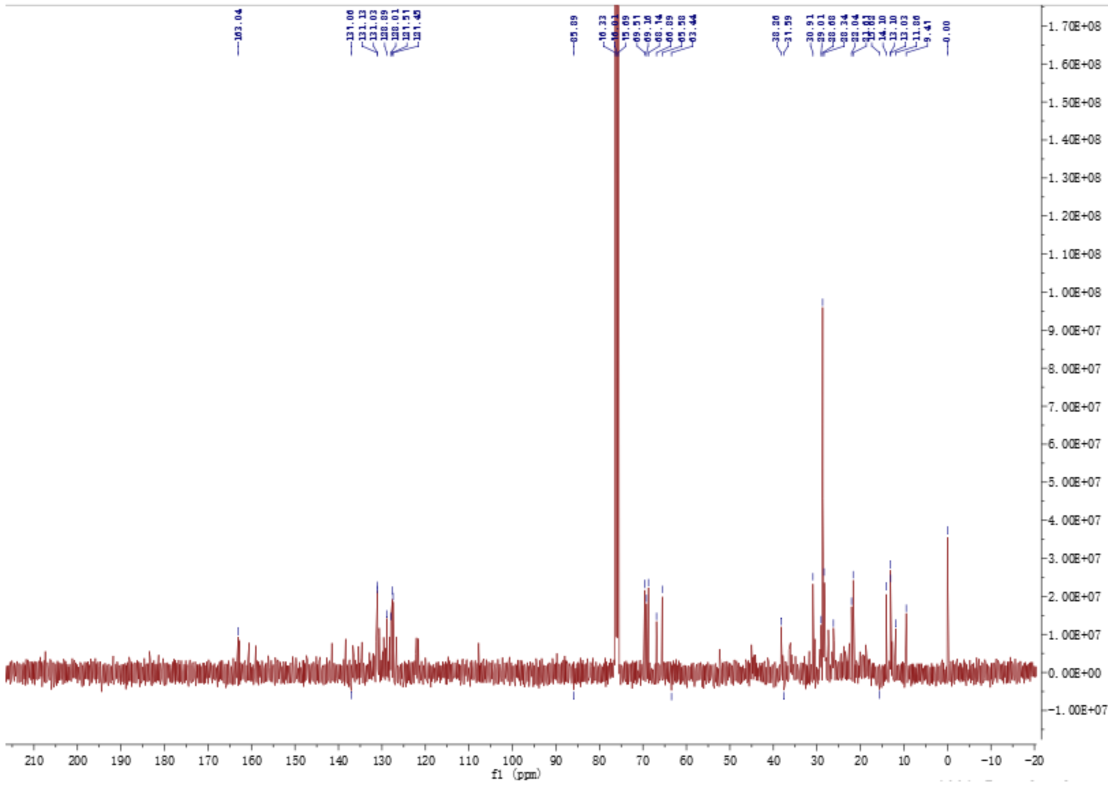


**Figure S13**. $^{13}C$ NMR spectrum of compound DPBDP

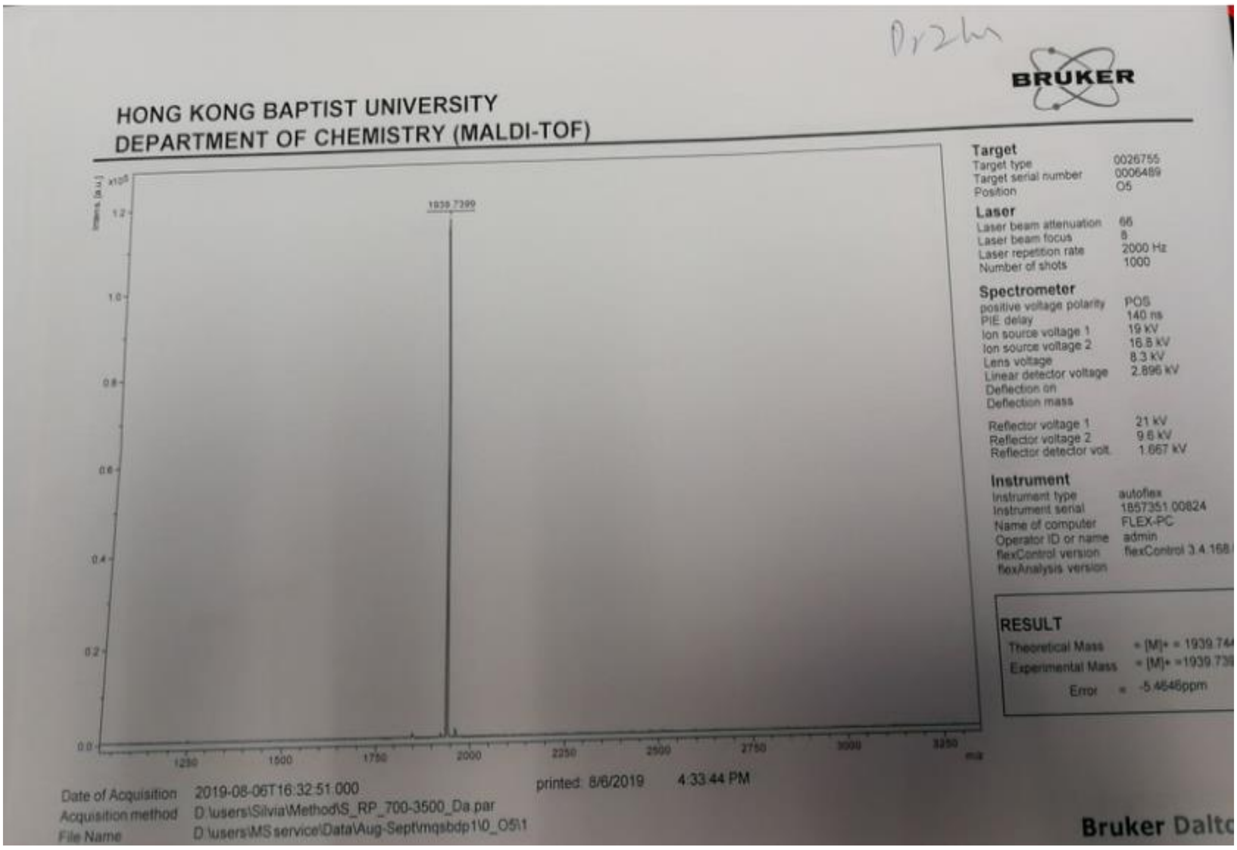


**Figure S14**. MALDI-TOF spectrum of compound DPBDP
